# Net health benefit of mavacamten for the treatment of Chinese patients with obstructive hypertrophic cardiomyopathy: a model-based economic evaluation

**DOI:** 10.3389/fphar.2025.1636732

**Published:** 2025-10-31

**Authors:** Han Bao, Yu Jia, Xingzhi Wang, Michael Hurst, Zoe Cheah, Melissa Ho, Jianwei Xuan

**Affiliations:** ^1^ Sun Yat-Sen University, Guangzhou, China; ^2^ Bristol Myers Squibb, Shanghai, China; ^3^ Bristol Myers Squibb, Uxbridge, United Kingdom; ^4^ OPEN Health HEOR & Market Access, London, United Kingdom

**Keywords:** net health benefits, obstructive HCM, hypertrophic cardiomyopathy, economic evaluation, mavacamten

## Abstract

**Objective:**

Obstructive hypertrophic cardiomyopathy (HCM) is a cardiovascular disorder characterized by significant symptom burden. This study aims to evaluate the net clinical benefit of mavacamten (a first-in-class cardiac myosin inhibitor) ± beta-blockers/calcium channel blockers (BB/CCBs) compared to BB/CCB monotherapy for Chinese patients with obstructive HCM.

**Methods:**

A five-state Markov model (NYHA classes I–IV and death) was developed from a Chinese healthcare system perspective. Patients were initialised in NYHA II or III states, mirroring the baseline distribution in the EXPLORER-CN trial (NCT05174416). State transitions were simulated using cycle-specific probabilities derived from trial data and validated extrapolation assumptions. All-cause mortality risks incorporated disease-specific and extra surgical mortality rates. Treatment sequencing rules reflected escalation pathways (e.g., non-response, adverse events, or eligibility for septural reduction therapy [SRT]), informed by Chinese clinical practice. Health utilities were mapped algorithmically from EXPLORER-CN patient-level EQ-5D responses.

**Results:**

Over a lifetime horizon, mavacamten ± BB/CCBs demonstrated superior health outcomes versus BB/CCB monotherapy, with incremental gains in life-years (15.76 vs. 14.40) and quality-adjusted life-years (QALYs: 13.69 vs. 11.38).

**Conclusion:**

Mavacamten provides substantial health benefits for Chinese obstructive HCM patients, including clinically meaningful improvements in survival and quality-of-life metrics relative to standard care.

## Introduction

Hypertrophic cardiomyopathy (HCM) is an often genetic, autosomal dominant myocardial disease classified into obstructive and non-obstructive based on the left ventricular outflow tract gradient (LVOTG) HCM can lead to sudden cardiac death or severe cardiovascular complications, significantly affecting patients’ daily lives and work (The Joint Committee of Cardiomyopathy Specialty Alliance, 2023). Obstructive HCM disproportionately affects younger populations, though substantial undiagnosed cases persist due to variable phenotypic expression (Burns and Jean-Pierre, 2018). Obstructive HCM poses a substantial risk, with a high likelihood of syncope and sudden cardiac death (Li et al., 2018).

Prior to the introduction of cardiac myosin inhibitors, treatment options for symptomatic obstructive HCM were pharmacological and invasive therapies. Pharmacological therapies include beta-blockers (BB) and calcium channel blockers (CCB) (The Joint Committee of Cardiomyopathy Specialty Alliance, 2023). These are not disease-specific treatments for obstructive HCM. While they can provide some symptom relief, they are generally ineffective in controlling the LVOT gradient and symptoms and cannot effectively and consistently relieve LVOT obstruction. Moreover, patients often do not receive adequate treatment or have poor tolerance (The Joint Committee of Cardiomyopathy Specialty Alliance, 2023; Ommen et al., 2020). Septural reduction therapy (SRT) are also a therapeutic option for eligible patients demonstrating New York Heart Association (NYHA) class III/IV symptoms or class II with exertion-induced syncope. SRT, which encompasses surgical myectomy, alcohol septal ablation, or electrophysiological ablation (radiofrequency septal ablation), effectively reduces LVOTG and improves symptoms (The Joint Committee of Cardiomyopathy Specialty Alliance, 2023). However, procedural success appears closely linked to surgical expertise and institutional experience, potentially limiting accessibility for regions with limited healthcare resources. Furthermore, while SRT provides therapeutic benefits, it should be noted that this intervention does not address the underlying pathophysiology of the disease. Given the progressive nature of the condition, recurrent obstructions may develop over time, potentially necessitating additional interventions to maintain clinical efficacy.

As a first-in-class small-molecule cardiac myosin inhibitor, mavacamten has been found to significantly reduce the LVOT gradient in obstructive HCM patients, effectively alleviate excessive myocardial contraction, enhance exercise capacity and quality of life, and markedly improve NYHA functional classification (Olivotto et al., 2020; Garcia-Pavia et al., 2024; Tian et al., 2023; Desai et al., 2021). Mavacamten has also demonstrated potential disease-modifying effects, improved cardiac structure, and significantly reduced the need for SRT. The safety and efficacy of mavacamten has been demonstrated in several key clinical trials, including: 1. The pivotal EXPLORER-HCM trial (NCT03470545), a global, multicentre, double-blind study designed to evaluate the efficacy and safety of mavacamten over 30 weeks in symptomatic (NYHA II/III) adult obstructive HCM patients (Olivotto et al., 2020). Compared to placebo, patients showed significant improvements in peak oxygen consumption, reduction in LVOT gradient, and NYHA functional classification. 2. The EXPLORER-LTE cohort of the MAVA-LTE 5-year extension open-label trial, following adults who completed EXPLORER-HCM. Its primary outcome was to assess the long-term safety and tolerability of mavacamten (Garcia-Pavia et al., 2024). 3. EXPLORER-CN (NCT05174416, sample size 81, 54 in the mavacamten group and 27 in the control group), a phase III, randomized, double-blind, placebo-controlled trial conducted in China, assessing the efficacy and safety of mavacamten compared to placebo in symptomatic obstructive HCM (NYHA II-III) adult patients with an LVOT gradient ≥50 mmHg at rest or after the Valsalva maneuverer (Tian et al., 2023). 4. VALOR-HCM (NCT04349072) is a double-blind RCT designed to explore the effect of mavacamten on reducing the need for SRT in patients with symptomatic obstructive HCM, the effect of mavacamten on SRT status, NYHA functional classification, etc (Desai et al., 2021).

Based on clinical trial evidence, a Markov model was constructed in Microsoft^®^ Excel to evaluate the long-term clinical benefits of mavacamten, either alone or in combination with BB or CCB monotherapy, compared to placebo (with or without BB or CCB monotherapy). The model examined Chinese adult patients with symptomatic obstructive HCM (NYHA II/III), focusing on life-years (LYs) and quality-adjusted life years (QALYs) gained relative to placebo from the perspective of the Chinese healthcare system, with 5% annual utility discounting rate.

## Materials and methods

### Model structure

A 5-state NYHA classification Markov model was constructed to assess the economic impact of mavacamten. There were several reasons for using the NYHA functional classification to define health states in published models (The National Institute for Health and Care Excellence NICE, 2023; The Canadian Agency for Drugs & Technologies in Health CADTH, 2023; Desai et al., 2022; Wasfy et al., 2021). First, NYHA classification is commonly used in clinical practice to assess symptoms and physical activity limitations in HCM patients. Second, it was a part of the primary endpoint (and a stand-alone secondary endpoint) in the EXPLORER-HCM trial and one of the key parameters for defining the composite primary endpoint in the EXPLORER-CN trial. Additionally, NYHA classification is an important predictor of all-cause and cardiovascular mortality in HCM patients, making it relevant to long-term survival (Desai et al., 2022). This model structure has been well-validated and is widely applied in economic evaluations in other cardiovascular fields such as heart failure with preserved/reduced ejection fraction (HFpEF/HFrEF) (Di Tanna et al., 2019; The National Institute for Health and Care Excellence NICE, 2021).

**FIGURE 1:**
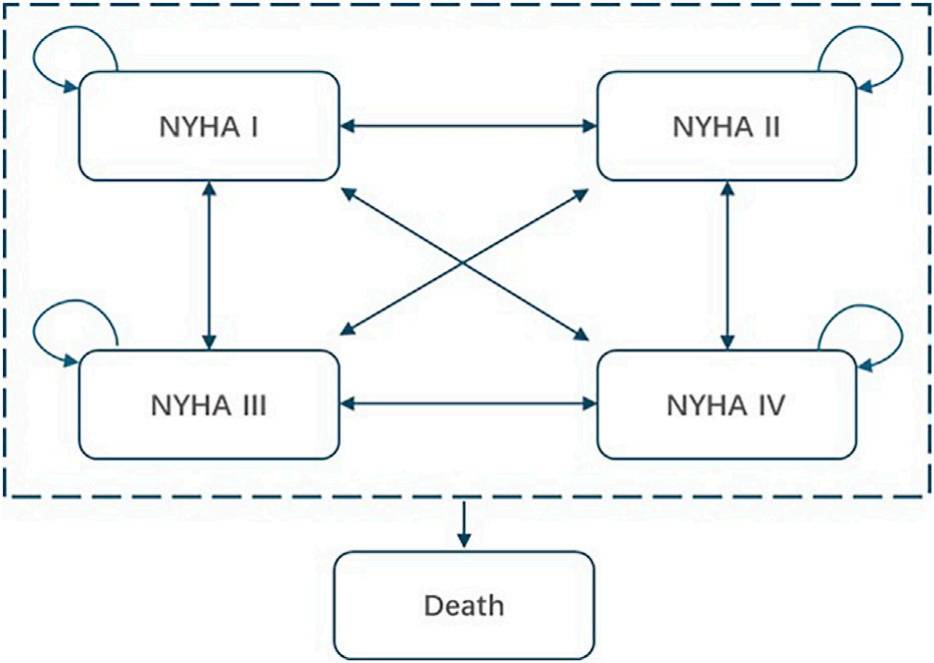
Simplified model structure of mavacamten for the treatment of obstructive HCM. NYHA, New York heart Association.

As shown in Figure 1, the simplified model structure categorized health states based on NYHA classification (NYHA I -IV) and death. All patients who entered the model were NYHA II or III, which was consistent with the baseline population distribution in the EXPLORER-CN trial and the regulatory label for mavacamten in China. In each simulation cycle, patients experienced improvements or deteriorations in their NYHA class, transitioning to different NYHA health states or remaining in the same state, based on the transition probabilities applied in the model corresponding to different interventions. Additionally, in each model cycle, all patients were considered to face a risk of death (both in terms of all-cause and cardiovascular [CV] related mortality).

### Cycle lengths and time horizon

During the first 30 weeks of the model (the clinical trial phase), cycle lengths were aligned with the clinical assessment time points of the EXPLORER-CN trial. This allowed the state transition probability matrix observed in the trial to be directly applied in the model, reflecting the actual effects of the clinical trial. After week 30, the model adopted a 4-week cycle length to align with the expected dosing regimen of mavacamten (28-day), enabling a sufficiently detailed simulation to accurately represent the drug’s benefits. To fully account for all costs and utility benefits associated with treatment, this study simulated the disease progression of patients over their lifetime.

### Patient treatment pathway

To accurately reflect clinical practice, and based on the recommendations of clinical experts, the model simulated patient transitions to various subsequent treatments. The simulated patient’s starting age is 42 years, which aligned with the average age of affected obstructive HCM patients (Burns and Jean-Pierre, 2018). The population baseline characteristics were based on the EXPLORER-CN trial, including the male-to-female ratio (male, 71.60% [95% CI 56.62%, 84.47%]) which was used to derive the weighted all-cause mortality rate (Tian et al., 2023). The proportion of patients within each NYHA class was used to initially distribute patients across different health states (NYHA functional class II, 76.54% [95% CI 60.03%, 89.68%]; NYHA functional class III, 23.46% [95% CI 19.02%, 28.20%]).

In the model, patients initially receiving mavacamten (with/without BB/CCB) would subsequently transition to BB/CCB monotherapy. Those maintained on BB/CCB monotherapy could then escalate to SRT alongside BB/CCB. Patients who received SRT were modelled for a single cycle and subsequently reverted back to a BB/CCB monotherapy treatment in the subsequent cycle and therein. During the first 30 weeks, all patients remained on their initial treatment plan. However, at the end of week 30 and at the start of each subsequent model cycle, patients could stop the initial treatment or switch to another therapy due to lack of response or severe adverse events (SAE, including Left ventricular ejection fraction [LVEF] <50%).

**FIGURE 2:**
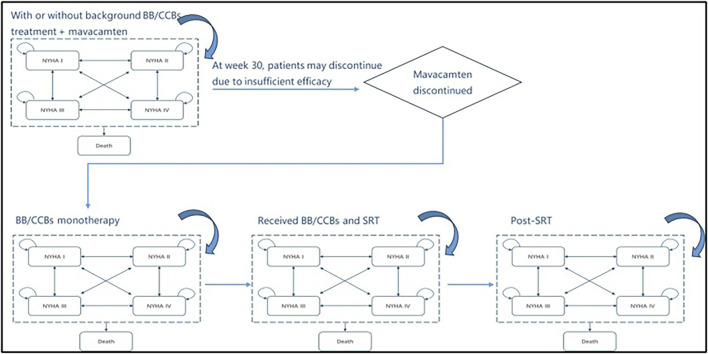
The simulation of the treatment pathway in the model. NYHA, New York Heart Association; BB/CCB, beta-blockers and calcium channel blockers; SRT, septural reduction therapy.

Escalation to (and choice of) subsequent treatment was driven by both response and adverse events derived from the EXPLORER-CN trial. Figure 2 illustrates the detailed simulation of the treatment pathway in the model. Similarly, the treatment switching rules applied to patients in the control group as well. SRT surgery was treated as an event in the model and patients with SRT treatment transitioned to a “post-SRT treatment” state after one cycle. The SRT tunnel stage was used to simulate the one-time costs, additional mortality risks associated with the surgery, and changes in NYHA health status resulting from the surgery. In the post-SRT state, patients essentially returned to BB/CCB monotherapy.

### Discontinuation and switching rules

There were two main aspects to the mavacamten discontinuation rule: 1. Discontinuation due to SAEs. The EXPLORER-CN trial demonstrated good safety, with no cases of treatment discontinuation due to SAEs. However, for the long-term simulation beyond the trial period, the probability of discontinuation due to SAEs was estimated at 5.00% per year. This physician-reported data was derived from a clinical physician survey in China, which involving 29 grade III hospitals treating over 3,000 HCM cases. The investigation comprised a qualitative phase with thematic analysis of 5 cardiologist in-depth interviews, followed by a quantitative phase administering 30 validated online questionnaires (15, data on file). 2. Discontinuation due to lack of treatment response. In the short-term phase of the model (up to week 30), the discontinuation rule was applied based on the EXPLORER-CN trial. In the long-term simulation (post week-30), only those patients who experienced worsening in their NYHA classification compared to the previous cycle were assumed to discontinue mavacamten, reflecting the likelihood of discontinuation due to worsening symptom burden.

Conversely, patients receiving BB/CCB monotherapy were at risk of requiring annual SRT surgery due to insufficient treatment efficacy, which led to switching to SRT surgery (Table 1). This estimate also came from the clinical physician survey in China.

**TABLE 1:** Physician-reported transition proportion from BB/CCB monotherapy to SRT (Tian et al., 2024) (annual, post-week 30, data on file).

NYHA I	NYHA II	NYHA III	NYHA IV
16.37% (95% CI 10.68%, 22.06%)	21.40% (95% CI 14.66%, 28.14%)	32.80% (95% CI 24.86%, 40.74%)	39.26% (95% CI 30.10%, 48.41%)

NYHA, new york heart association.

### Short-term TPs between health states

The short-term simulation aimed to fully reflect the clinical trial efficacy and was based entirely on individual patient-level data from the EXPLORER-CN trial, which tracked patient outcomes at various time points. The short-term state transition probability matrix (Table 2) was constructed from this data (individual-level calculations, not publicly disclosed).

**TABLE 2:** Model inputs: short-term TPs (based on EXPLORER-CN (Tian et al., 2023))^a^.

From/To	NYHA I	NYHA II	NYHA III	NYHA IV
Treatment group (with or without BB/CCB + mavacamten)				
*Baseline to Week 4*				
NYHA I	NA^a^	NA	NA	NA
NYHA II	0.0227	0.9773	0.0000	0.0000
NYHA III	0.0000	0.3000	0.7000	0.0000
NYHA IV	NA	NA	NA	NA
*Week 4 to Week 6*				
NYHA I	1.0000	0.0000	0.0000	0.0000
NYHA II	0.0000	1.0000	0.0000	0.0000
NYHA III	0.0000	0.1429	0.8571	0.0000
NYHA IV	NA	NA	NA	NA
*Week 6 to Week 8*				
NYHA I	1.0000	0.0000	0.0000	0.0000
NYHA II	0.0426	0.9362	0.0213	0.0000
NYHA III	0.0000	0.1667	0.8333	0.0000
NYHA IV	NA	NA	NA	NA
*Week 8 to Week 12*				
NYHA I	1.0000	0.0000	0.0000	0.0000
NYHA II	0.0667	0.9333	0.0000	0.0000
NYHA III	0.0000	0.3333	0.6667	0.0000
NYHA IV	NA	NA	NA	NA
*Week 12 to Week 14*				
NYHA I	1.0000	0.0000	0.0000	0.0000
NYHA II	0.0227	0.9318	0.0455	0.0000
NYHA III	0.0000	0.0000	1.0000	0.0000
NYHA IV	NA	NA	NA	NA
*Week 14 to Week 18*				
NYHA I	1.0000	0.0000	0.0000	0.0000
NYHA II	0.0244	0.9756	0.0000	0.0000
NYHA III	0.0000	0.3333	0.6667	0.0000
NYHA IV	NA	NA	NA	NA
*Week 18 to Week 20*				
NYHA I	1.0000	0.0000	0.0000	0.0000
NYHA II	0.0000	1.0000	0.0000	0.0000
NYHA III	0.0000	0.0000	1.0000	0.0000
NYHA IV	NA	NA	NA	NA
*Week 20 to Week 24*				
NYHA I	1.0000	0.0000	0.0000	0.0000
NYHA II	0.0952	0.8810	0.0238	0.0000
NYHA III	0.0000	0.0000	1.0000	0.0000
NYHA IV	NA	NA	NA	NA
*Week 24 to Week 26*				
NYHA I	1.0000	0.0000	0.0000	0.0000
NYHA II	0.0000	1.0000	0.0000	0.0000
NYHA III	0.0000	0.0000	1.0000	0.0000
NYHA IV	NA	NA	NA	NA
*Week 26 to Week 30*				
NYHA I	1.0000	0.0000	0.0000	0.0000
NYHA II	0.3243	0.6757	0.0000	0.0000
NYHA III	0.0000	0.4000	0.6000	0.0000
NYHA IV	NA	NA	NA	NA

Control group (with or without BB/CCB monotherapy)				
*Baseline to Week 4*				
NYHA I	NA	NA	NA	NA
NYHA II	0.0000	1.0000	0.0000	0.0000
NYHA III	0.0000	0.1111	0.8889	0.0000
NYHA IV	NA	NA	NA	NA
*Week 4 to Week 6*				
NYHA I	NA	NA	NA	NA
NYHA II	0.0000	1.0000	0.0000	0.0000
NYHA III	0.0000	0.1250	0.8750	0.0000
NYHA IV	NA	NA	NA	NA
*Week 6 to Week 8*				
NYHA I	NA	NA	NA	NA
NYHA II	0.0000	0.9474	0.0526	0.0000
NYHA III	0.0000	0.0000	1.0000	0.0000
NYHA IV	NA	NA	NA	NA
*Week 8 to Week 12*				
NYHA I	NA	NA	NA	NA
NYHA II	0.0556	0.8889	0.0556	0.0000
NYHA III	0.0000	0.1429	0.8571	0.0000
NYHA IV	NA	NA	NA	NA
*Week 12 to Week 14*				
NYHA I	1.0000	0.0000	0.0000	0.0000
NYHA II	0.0000	0.9412	0.0588	0.0000
NYHA III	0.0000	0.0000	1.0000	0.0000
NYHA IV	0.0000	0.0000	0.0000	1.0000
*Week 14 to Week 18*				
NYHA I	1.0000	0.0000	0.0000	0.0000
NYHA II	0.0000	1.0000	0.0000	0.0000
NYHA III	0.0000	0.2500	0.7500	0.0000
NYHA IV	NA	NA	NA	NA
*Week 18 to Week 20*				
NYHA I	1.0000	0.0000	0.0000	0.0000
NYHA II	0.0000	1.0000	0.0000	0.0000
NYHA III	0.0000	0.0000	1.0000	0.0000
NYHA IV	NA	NA	NA	NA
*Week 20 to Week 24*				
NYHA I	1.0000	0.0000	0.0000	0.0000
NYHA II	0.0000	1.0000	0.0000	0.0000
NYHA III	0.0000	0.1667	0.8333	0.0000
NYHA IV	NA	NA	NA	NA
*Week 24 to Week 26*				
NYHA I	1.0000	0.0000	0.0000	0.0000
NYHA II	0.0000	1.0000	0.0000	0.0000
NYHA III	0.0000	0.0000	1.0000	0.0000
NYHA IV	NA	NA	NA	NA
*Week 26 to Week 30*				
NYHA I	1.0000	0.0000	0.0000	0.0000
NYHA II	0.0000	1.0000	0.0000	0.0000
NYHA III	0.0000	0.0000	1.0000	0.0000
NYHA IV	NA	NA	NA	NA

^a^ EXPLORER-CN trial (sample size 81); NA, refers to no one being in the health state of interest; 95% CI, was unavailable, joint parameter uncertainty was propagated via PSA, using Dirichlet distributions.

NYHA, new york heart association; BB/CCB, beta-blockers and calcium channel blockers.

### Long-term TPs between health states

The long-term state transition probability was extrapolated based on clinicians’ estimates and patient health trends observed during the trial period. Existing literature did not provide evidence of any significant effect of standard treatments on disease progression. The EXPLORER-CN trial also showed that patients in the control (placebo + BB/CCB monotherapy) group gradually exhibited a trend of being unable to sustain health improvements observed prior to week 18 (Tian et al., 2023). Therefore, the model assumes that, in the control group, patients would not undergo NYHA functional class improvement in the long-term simulation unless they switched to other therapies.

In the EXPLORER-LTE cohort of the MAVA-LTE trial, patients’ reported NYHA health status steadily improved between 48 and 108 weeks post-clinical trial (Garcia-Pavia et al., 2024), supporting the model’s conservative assumption of sustained intervention-induced health state improvements persisting through to at least week 108. Therefore, mavacamten patients in the EXPLORER-CN trial with health status improvements during weeks 26–30 would maintain progressive NYHA class optimization beyond week 30. Based on the 4-week simulation cycle of the model, the 108-week period was adjusted to 106 weeks in accordance with the actual situation of the model.

In summary, for the mavacamten group, the health state transition probability was derived from the clinical trial results of week 0–30 and extrapolate for week 30–106 (Table 3). After week 106, a conservative assumption was made where no further health state transitions occurreds. For the control group, the state transition probability matrix derived from the clinical trial results of week 0–30. After week 30, it was assumed that patients did not experience any health-state transitions.

**TABLE 3:** Model inputs: per cycle long-term TPs and TPs adjusted by natural disease progression.

From/To	NYHA I	NYHA II	NYHA III	NYHA IV
Treatment group (with or without BB/CCB + mavacamten)				
*30 weeks - 106 weeks (Inspired by EXPLORER-LTE* (Garcia-Pavia et al., 2024)*)*				
NYHA I	1.0000	0.0000	0.0000	0.0000
NYHA II	0.3243	0.6757	0.0000	0.0000
NYHA III	0.0000	0.4000	0.6000	0.0000
NYHA IV	0.0000	0.0000	0.0000	1.0000
*>106 weeks (based on natural disease progression)*				
NYHA I	0.9966	0.0034	0.0000	0.0000
NYHA II	0.0000	0.9966	0.0034	0.0000
NYHA III	0.0000	0.0000	0.9966	0.0034
NYHA IV	0.0000	0.0000	0.0000	1.0000

Control group (with or without BB/CCB monotherapy)				
*>30 weeks (based on natural disease progression)*				
NYHA I	0.9966	0.0034	0.0000	0.0000
NYHA II	0.0000	0.9966	0.0034	0.0000
NYHA III	0.0000	0.0000	0.9966	0.0034
NYHA IV	0.0000	0.0000	0.0000	1.0000

The upper and lower limits of variables in natural disease progression were adjusted based on the 95% CI, of the weighted average annual progression rate of 4.55% (95% CI, 3.70%, 5.48%).

NYHA, new york heart association; BB/CCB, beta-blockers and calcium channel blockers.

### Adjustment for natural disease progression

Although no NYHA class IV patients were observed in the EXPLORER-CN trial, based on clinical realities iof HCM as a progressive disease and the advice of healthcare professionals, the model incorporated the impact of NYHA IV patients to more accurately reflect the long-term symptom burden. The model adjusted the long-term state transition probability matrix to account for the natural deterioration of NYHA class due to disease duration and aging. A weighted average annual progression rate of 4.55% (95% CI 3.70%, 5.48%) was used in the model (Table 3) (Maron et al., 2016; Maron et al., 2018). Consequently, the model used a natural disease progression matrix to further adjust the assumption of no health state transitions, considering that patients might experience deterioration due to natural disease progression.

### Surgical efficacy

As mentioned prior, the model used different state transition probability matrices to demonstrate the treatment effects of SRT and health status post-surgery, where the post-surgery state transition probability matrix was identical to that of patients receiving BB/CCB monotherapy alone. The efficacy of SRT was based on a retrospective observational study involving 752 patients diagnosed with obstructive HCM (Table 4) (Barriales-Villa et al., 2025).

**TABLE 4:** Model inputs: surgical efficacy and post-SRT efficacy.

From/To	NYHA I	NYHA II	NYHA III	NYHA IV
BB/CCBs + SRT (applied for single cycle, Barriales-Villa R. et al. (Barriales-Villa et al., 2025))				
NYHA I	1.0000	0.0000	0.0000	0.0000
NYHA II	0.0000	1.0000	0.0000	0.0000
NYHA III	0.0000	0.3871	0.6129	0.0000
NYHA IV	0.0000	0.0000	0.3548	0.6452
Post-SRT (applied per cycle)				
NYHA I	0.9966	0.0034	0.0000	0.0000
NYHA II	0.0000	0.9966	0.0034	0.0000
NYHA III	0.0000	0.0000	0.9966	0.0034
NYHA IV	0.0000	0.0000	0.0000	1.0000

95% CI, was unavailable, joint parameter uncertainty was propagated via PSA, using Dirichlet distributions.

NYHA, new york heart association; BB/CCB, beta-blockers and calcium channel blockers; SRT, septural reduction therapy.

### Mortality

The all-cause mortality data for the general population was derived from a life table using mortality data from China’s seventh national census, which reflected the average mortality rate of the population. The life table was then adjusted to reflect the sex-distribution of the population modelled (Office of the Leading Group of the Stat e Council for the Seventh National Population Census, 2021). CV-related mortality was based on an analysis with health record database in the United States (Wang et al., 2023). The relative risks for CV-related mortality per NYHA class (Table 5) were applied to the underlying all-cause mortality with NYHA class I acting as a reference category, assuming that a NYHA class I patients’ mortality was equal to that of the general population.

**TABLE 5:** Model inputs: hazard ratios for excess mortality by NYHA state (Wang et al., 2023).

NYHA I	NYHA II vs. I	NYHA III vs. I	NYHA IV vs. I
Reference (1.00) (95% CI 0.82, 1.21)	1.80 (95% CI 1.40, 2.32)	4.12 (95% CI 3.24, 5.25)	10.90 (95% CI 8.28, 14.35)

NYHA, new york heart association.

In addition to mortality risk differences based on NYHA classes, the model also considered the impact of a single SRT procedure on mortality risks. A series of published data was collected and analyzed to calculate the weighted mortality rate associated with SRT surgery (∼1.80%; calculation process shown in Supplementary Table S1).

### Utility

The EXPLORER-CN trial did not collect EQ-5D-5L scale data, while the disease-specific patient-reported outcomes using the Kansas City Cardiomyopathy Questionnaire-23 (KCCQ-23) was collected. The study used the mapping relationship between the KCCQ-23 scores and the Chinese EQ-5D-5L utility index system (Cheah et al., 2024; Luo et al., 2017). By incorporating individual KCCQ-23 domain score data from the EXPLORER-CN trial, utility values for the Chinese population in NYHA I/II/III states were adjusted accordingly (Table 6). The utility analyses showed treatment arm and NYHA class highly correlated and the utility values were further applied in the model irrespective of treatment received.

**TABLE 6:** Utility values for patients in different NYHA states (Cheah et al., 2024).

NYHA I	NYHA II	NYHA III	NYHA IV
0.905 (95% CI 0.89, 0.91)	0.845 (95% CI 0.84, 0.85)	0.687 (95% CI 0.66, 0.71)	0.687 (95% CI 0.66, 0.71)

NYHA, new york heart association; NYHA IV, utility is assumed equal to NYHA III, utility.

### Sensitivity analysis

The study conducted a one-way deterministic sensitivity analysis (DSA) to identify the model parameters that have the greatest impact on the results and to illustrate the lower-value versus higher-value scenarios. Each parameter was assigned to “lower” and “upper” values based on its 95% confidence interval (CI). When a 95% CI was unavailable, the standard error was assumed to be 10% of the point estimate to generate the upper and lower limits. The results were presented in tornado plots, highlighting the parameters that contributed most to the uncertainty in the model’s results. The study also performed a probabilistic sensitivity analysis (PSA) on key parameters. Monte Carlo simulations were used to draw values from the individual uncertainty distributions of each parameter, with the chosen distributions reflecting the known upper and lower limits of the parameters. PSA allows all model parameters to vary simultaneously within a reasonable range. To ensure the stability of the results, PSA was performed with 5,000 iterations of the patient cohort.

## Results

The deterministic analysis revealed that treatment with mavacamten for adult obstructive HCM yields an average of 15.76 LYs and 13.69 QALYs gained per patient over a lifetime after 5% discount annually. In comparison, standard therapy (monotherapy with BB/CCB) was projected to result in 14.40 LYs and 11.38 QALYs, resulting in an incremental gain of around 1.37 LYs and 2.31 QALYs.

Patients receiving mavacamten, either alone or in combination with BB/CCB monotherapy, were estimated to experience 9.67 additional life years in NYHA functional class I (10.11 years compared to 0.44 years), 4.64 fewer life years in NYHA II (4.19 years versus 8.83 years), 2.78 fewer in NYHA III (1.20 years versus 3.98 years) and 0.89 fewer in NYHA IV (0.26 years versus 1.15 years) compared to those treated in the control group (Table 7). This was driven primarily by the improved NYHA distribution of EXPLORER-CN. When considering QALYs gained, patients in the treatment group were estimated to spend 1.69 additional QALYs in NYHA I, 0.81 higher in NYHA II, 0.04 higher in NYHA III, and 0.22 fewer in NYHA IV compared to those received BB/CCBs monotherapy.

**TABLE 7:** Deterministic LYs and QALYs; overall and by NYHA class.

Intervention	Overall	NYHA I	NYHA II	NYHA III	NYHA IV
LYs					
Treatment group	15.76	10.11	4.19	1.20	0.26
Control group	14.40	0.44	8.83	3.98	1.15
**Difference**	**+1.37**	**+9.67**	**−4.64**	**−2.78**	**−0.89**
QALYs					
Treatment group	13.69	9.15	3.54	0.83	0.18
Control group	11.38	7.46	2.73	0.79	0.40
**Difference**	**+2.31**	**+1.69**	**+0.81**	**+0.04**	**−0.22**

NYHA, new york heart association; LYs, Life-years; QALYs, quality-adjusted life years. Bold values indicates the difference of treatment group - control group.

**FIGURE 3:**
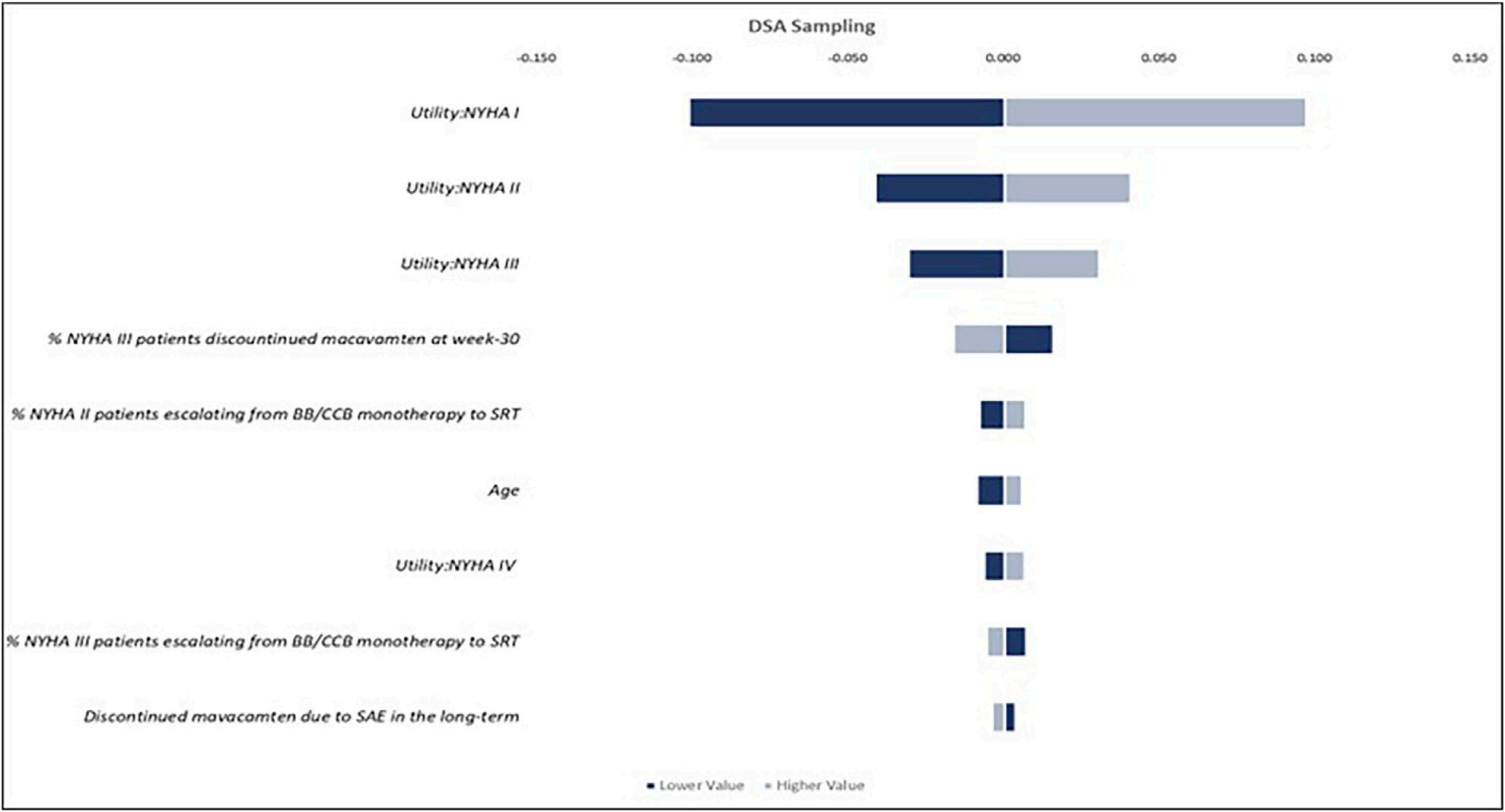
One‐way DSA results.

The results of the one-way DSA indicated that after comparing lower-value versus higher-value scenarios of the ICERs calculated based on the confidence intervals of different parameters, the utility values assigned to patients in NYHA functional classes I, II, and III (Figure 3), the proportion of patients discontinuing mavacamten at week 30 while remaining in NYHA class III, and the proportion of patients escalated from BB/CCB monotherapy to SRT, had the largest impact on QALYs, among others. The PSA, conducted using 5,000 Monte Carlo simulations, showed that the incremental value of LYs and QALYs were closely aligned with the results of the base-case analysis, with the PSA mean yielding an increase for mavacamten of 1.363 (95% CI 1.363, 1.364) and 2.310 (95% CI 2.307, 2.312) for incremental LYs and QALYs, respectively, demonstrating the robustness of the model.

## Discussion

This study developed a Markov model based on the NYHA classification to evaluate the long-term benefits of mavacamten compared to BB/CCB monotherapy for adult patients with obstructive HCM. From the perspective of the Chinese healthcare system, the results indicated significant advantages in both life years and QALYs for patients using mavacamten. Lifetime treatment with mavacamten was estimated to provide an additional 1.37 life years and 2.31 QALYs (discounted at 5% annually) compared to BB/CCB monotherapy. PSA yielded results consistent with the base-case analysis, demonstrating the model’s robustness.

Some assumptions in the study were conservative. For example, the study did not account for potential long-term adverse effects or monitoring after SRT due to limited evidence. Given the anticipated higher incidence of SRT among patients on BB/CCB monotherapy, this conservative approach might have underestimated the disutility associated with BB/CCB treatment. Furthermore, the model might underestimate QALY gains. In this therapeutic area, unmet needs were substantial. Patients with NYHA class II/III often made significant lifestyle adjustments in the absence of effective treatments. The introduction of new therapies could lead to lifestyle improvements that might not have been fully captured by the EQ-5D scale.

This study has several strengths. The model was constructed using trial data specific to Chinese patients, and most of the referenced literature reflects the treatment landscape of this disease area, ensuring high representativeness. The model also incorporated actual treatment pathways and patterns observed in Chinese clinical practice. However, the study has limitations. First, the extrapolation of lifetime outcomes based on short-term clinical trial data represents a significant constraint. Specifically, the assumption that the efficacy of mavacamten lasts only 106 weeks—after which patients are assumed to either sustain the improved state and revert to natural progression—was derived from the currently limited follow-up data. While this conservative approach might lead to an underestimation of the treatment’s long-term benefits, it was deemed necessary in the absence of extended real-world evidence. Such assumptions were necessary to minimize decision-making risks and ensure methodological relevance. Potential long-term efficacy patterns, such as sustained response, waning effect, or loss of effect, remain uncertain and should be further investigated once longer-term data become available. Additionally, although this study focuses on a Chinese population, certain general parameters were informed by international literature due to limited local data, which may affect the generalizability of the findings. Long-term follow-up data on the natural progression of obstructive HCM and the efficacy of SRT in Chinese patients were currently unavailable, which may have introduced some inaccuracies into the analysis. Furthermore, since NYHA IV patients did not appear in Chinese clinical trials, utility values for this group could not be mapped from the trial. The assumption that NYHA class IV utility values were the same as those for NYHA class III might overestimate the benefits of the intervention in class IV, although this group represented a small proportion in the model.

## Conclusion

The findings of this study demonstrate that mavacamten offers substantial therapeutic advantages for Chinese patients with obstructive HCM. Compared to the BB/CCB monotherapy, mavacamten enhances long-term survival and improves QALY outcomes, positioning it as a transformative intervention in this underserved population.

## Data Availability

The datasets presented in this article are not readily available because This study used anonymous, processed and structured clinical trial data for analysis, and the data is not publicly available. Requests to access the datasets should be directed to HB, baoh9@mail.sysu.edu.cn.

## Abbreviations

BB, Beta-blockers; CCB, Calcium channel blockers; HCM, Hypertrophic cardiomyopathy; KCCQ-23, Kansas City Cardiomyopathy Questionnaire-23; LVOTG, Left ventricular outflow tract gradient; LVEF, Left ventricular ejection fraction; LYs, Life-years; NYHA, New York Heart Association; QALYs, Quality-adjusted life years; SRT, Septural reduction therapy; TP, Transition probability.
