# Supplementary material for: Net health benefit of mavacamten for the treatment of Chinese patients with obstructive hypertrophic cardiomyopathy: a model-based economic evaluation
Source: Front Pharmacol. 2025 Oct 31;16:1636732. doi: 10.3389/fphar.2025.1636732 (PMC12615438; doi:10.3389/fphar.2025.1636732)
Supplement: Supplementary file 1 [file Table1.docx]

**SUPPLEMENTARY I. Mortality risks associated with SRT**

| **Data source** | **Alcohol ablation therapy** | | **Myectomy** | |
| --- | --- | --- | --- | --- |
|  | **N (n)** | **Surgical mortality (%)** | **N (n)** | **Surgical mortality (%)** |
| Altibi et al. (23) | 6,942 (84) | 1.2% | 12,065 (482) | 4.0% |
| Mentias et al. (24) | 1,999 (34) | 1.7% | N/A | N/A |
| Kim et al. (25) | 4,862 (34) | 0.7% | 6,386 (332) | 5.2% |
| Maksabedian Hernandez et al. (26) | 1,393 (17) | 1.2% | 1,668 (63) | 3.8% |
| Mazine et al. (27) | N/A | N/A | 26 (0) | 0.0% |
| Pedernera et al. (28) | 23 (0) | 0.0% | N/A | N/A |
| Ullah et al. (29) | 5,235 (70) | 1.3% | N/A | N/A |
| Hadaya et al. (30) | N/A | N/A | 3,338 (112) | 3.4% |
| Fortunato et al. (31) | 56 (1) | 1.8% | N/A | N/A |
| Delgado et al. (32) | 30 (1) | 3.3% | N/A | N/A |
| Veselka et al. (33) | 100 (0) | 0.0% | N/A | N/A |
| Vresendorp et al. (34) | 321 (5) | 1.6% | 253 (3) | 1.2% |
| Yasuda et al. (35) | 348 (3) | 0.9% | 407 (14) | 3.4% |
| **Weighted** | **21,309 (249)** | **1.17%** | **24,143 (1,007)** | **4.17%** |

**SUPPLEMENTARY REFERENCES**

23. Altibi AM, Ghanem F, Zhao Y, et al. Hospital procedural volume and clinical outcomes following septal reduction therapy in obstructive hypertrophic cardiomyopathy. *J Am Heart Assoc*. (2023) 12:e028693. doi: 10.1161/JAHA.122.028693.

24. Mentias A, Smedira NG, Krishnaswamy A, et al. Survival after septal reduction in patients >65 years old with obstructive hypertrophic cardiomyopathy. *J Am Coll Cardiol*. (2023) 81:105-115. doi: 10.1016/j.jacc.2022.10.027.

25. Kim LK, Swaminathan RV, Looser P, et al. Hospital volume outcomes after septal myectomy and alcohol septal ablation for treatment of obstructive hypertrophic cardiomyopathy: US nationwide inpatient database, 2003-2011. *JAMA Cardiol*. (2016) 1:324-32. doi: 10.1001/jamacardio.2016.0252.

26. Maksabedian Hernandez E, Krishnaswami S, Dubey A, et al. Association between hospital volume, clinical events, resource utilization, and costs of septal myectomy and alcohol septal ablation procedures in US patients with hypertrophic cardiomyopathy. *European Heart Journal*. (2023) 44: Issue Supplement_2, ehad655.1853. doi:10.1093/eurheartj/ehad655.1853.

27. Mazine A, Ghoneim A, Bouhout I, et al. A novel minimally invasive approach for surgical septal myectomy. *Can J Cardiol*. (2016) 32:1340-1347. doi: 10.1016/j.cjca.2016.01.034.

28. Pedernera GO, Costabel JP, Avegliano G, et al. Clinical and functional outcome of percutaneous alcohol septal ablation in obstructive hypertrophic cardiomyopathy. *Rev. Argent. Cardiol.* (2015) 217-222. doi: 10.7775/rac.v83.i3.5919.

29. Ullah W, Warner E, Khandait H, et al. Septal myectomy or alcohol ablation for hypertrophic cardiomyopathy: a nationwide inpatient sample (NIS) database analysis. *Cardiovasc Revasc Med*. (2023) 50:54-58. doi: 10.1016/j.carrev.2023.01.013.

30. Hadaya J, Verma A, Sanaiha Y, et al. Volume-outcome relationship in septal myectomy for hypertrophic obstructive cardiomyopathy. *Surgery*. (2023) 174,166-171. doi: 10.1016/j.surg.2023.04.028.

31. Fortunato de Cano S, Nicolas Cano M, de Ribamar Costa J Jr, et al. Long-term clinical follow-up of patients undergoing percutaneous alcohol septal reduction for symptomatic obstructive hypertrophic cardiomyopathy. *Catheter Cardiovasc Interv.* (2016) 88:953-960. doi: 10.1002/ccd.26430.

32. Delgado V, Sitges M, Andrea R, et al. Seguimiento clínico y ecocardiográfico de pacientes con miocardiopatía hipertrófica obstructiva tratados con ablación septal percutánea [Clinical and echocardiographic follow-up of patients with hypertrophic obstructive cardiomyopathy treated by percutaneous septal ablation]. *Rev Esp Cardiol*. (2006) 59:1123-30. doi: 10.1157/13095781. [Article in Spanish].

33. Veselka J, Tomasov P, Zemánek D. Mid-term outcomes of alcohol septal ablation for obstructive hypertrophic cardiomyopathy in patients with sigmoid versus neutral ventricular septum. *J Invasive Cardiol.* (2012) 24:636-640. PMID: 23220977.

34. Vriesendorp PA, Liebregts M, Steggerda RC, et al. Long-term outcomes after medical and invasive treatment in patients with hypertrophic cardiomyopathy. *JACC Heart Fail*. (2014) 2:630-6. doi: 10.1016/j.jchf.2014.06.012.

35. Yasuda R, Osawa I, Goto T, et al. Mortality after alcohol septal ablation vs. septal myectomy in patients with obstructive hypertrophic cardiomyopathy. *Circ Rep.* (2024) 6:74-79. doi: 10.1253/circrep.CR-23-0101.
